# Optimizing Surgical Drain Removal: A Narrative Review of Timing, Criteria, and Evidence-Based Practices

**DOI:** 10.7759/cureus.94492

**Published:** 2025-10-13

**Authors:** Mark Salib, John Salib, Elias Kondilis, Almir Music, Matthew Phillips

**Affiliations:** 1 School of Medicine, St. George's University School of Medicine, St. George's, GRD; 2 General Surgery, Community First Medical Center, Chicago, USA

**Keywords:** criteria for drain removal, drain management, drain protocols, enhanced recovery after surgery (eras), fluid output, postoperative care, surgical complications, surgical drains, timing of drain removal

## Abstract

Surgical drains remain a common adjunct in general surgery to prevent fluid collections and detect early postoperative complications. However, their routine use and timing of removal continue to generate debate. This review analyzes evidence-based criteria for drain removal, identifies complications related to prolonged use, and evaluates evolving trends in drain management across gastrointestinal, hepatobiliary, pancreatic, and abdominal wall procedures. The evidence consistently supports early drain removal typically once drainage output is <50 mL/24 h and non-bilious or non-hemorrhagic being associated with lower rates of surgical site infection, fistula formation, and hospital stay duration. Conversely, delayed removal beyond postoperative day five to seven increases the risk of infection, prolonged inflammation, and wound complications. Biochemical monitoring, particularly amylase and bilirubin levels in pancreatic and biliary drains, enables safer, individualized removal decisions. Early, criteria-based, and selective drain management integrated within Enhanced Recovery After Surgery (ERAS) frameworks improves postoperative outcomes and reduces morbidity in general surgery. These findings emphasize the importance of tailored protocols and continued refinement of evidence-driven drain removal practices.

## Introduction and background

Surgical drains are commonly used to remove postoperative fluid accumulations such as blood, serous fluid, or lymph, helping to prevent complications and support proper wound healing [1]. Effective management of these drains, particularly the timing of their removal, is important to maximize their benefits while minimizing associated risks. The decision to remove a drain typically depends on the type of surgery performed, the volume and rate of fluid output, relevant biochemical markers, and the patient’s overall clinical condition [2,3]. When clinically appropriate, earlier drain removal guided by objective criteria, such as low output volume, favourable fluid analysis, or a defined postoperative timeline, has been shown to reduce the risks of infection, fistula formation, and extended hospital stays [4].

Prolonged drain placement, on the other hand, can lead to several complications, including surgical site infections, local tissue irritation, drain blockage or migration, bleeding, and delayed healing [5]. These situations may prolong hospitalization and require additional interventions. Different surgical fields have developed varying practices regarding drain removal, often based on fluid output thresholds and a defined postoperative timeline. In pancreatic surgery, for example, drain amylase levels are routinely used to guide timing, while in breast and head and neck surgery, decisions are often based on drainage volume over time [6,7]. Despite these differences, there is increasing recognition of the need for individualized, evidence-based protocols that combine quantitative data with clinical judgment. This approach reflects a broader effort to improve patient outcomes while avoiding unnecessary delays in recovery [8].

Although numerous studies have examined the use of drains in specific surgical subspecialties, the literature remains fragmented, with limited consensus on standardized removal criteria across general surgery. Current recommendations are often derived from small, procedure-specific cohorts or single-institution experiences, leading to inconsistent clinical practice. Furthermore, many Enhanced Recovery After Surgery (ERAS) guidelines reference drain use only indirectly, leaving uncertainty about optimal timing, biochemical thresholds, and clinical indicators for safe removal. This review seeks to bridge these gaps by synthesizing existing evidence into a unified, general surgery-specific framework emphasizing early, individualized, and outcome-oriented drain management.

This review examines current practices and evidence surrounding surgical drain removal across various procedures. It explores the criteria commonly used to guide timing, such as output volume, biochemical markers, and clinical assessment, and highlights variations across surgical specialties. By synthesizing available data and expert recommendations, the paper aims to clarify best practices and support more consistent, patient-centered drain management.

History and background

Surgical drains have been used since ancient times, with early descriptions by Hippocrates and Galen using hollow reeds and animal horns to evacuate pus or fluid, mainly for abscesses and traumatic injuries [9]. Their routine use in elective surgery was limited until the 19th century, when Lister’s antiseptic techniques enabled safer, more hygienic drains made of rubber and glass [10], which became common in abdominal and thoracic surgeries [11].

The mid-20th century introduced closed-suction systems like Jackson-Pratt and Hemovac drains, allowing continuous sterile drainage and improved mobility [9]. Advances in surgical technique and postoperative care led to more selective use, with attention to both placement and timely removal to reduce infection, fistula, and delayed healing [12]. Recent ERAS protocols and trial data in colorectal, breast, and thyroid surgery have further challenged routine drainage, emphasizing individualized, evidence-based decisions to optimize recovery and minimize complications [12-15].

## Review

Methods

**Figure 1:**
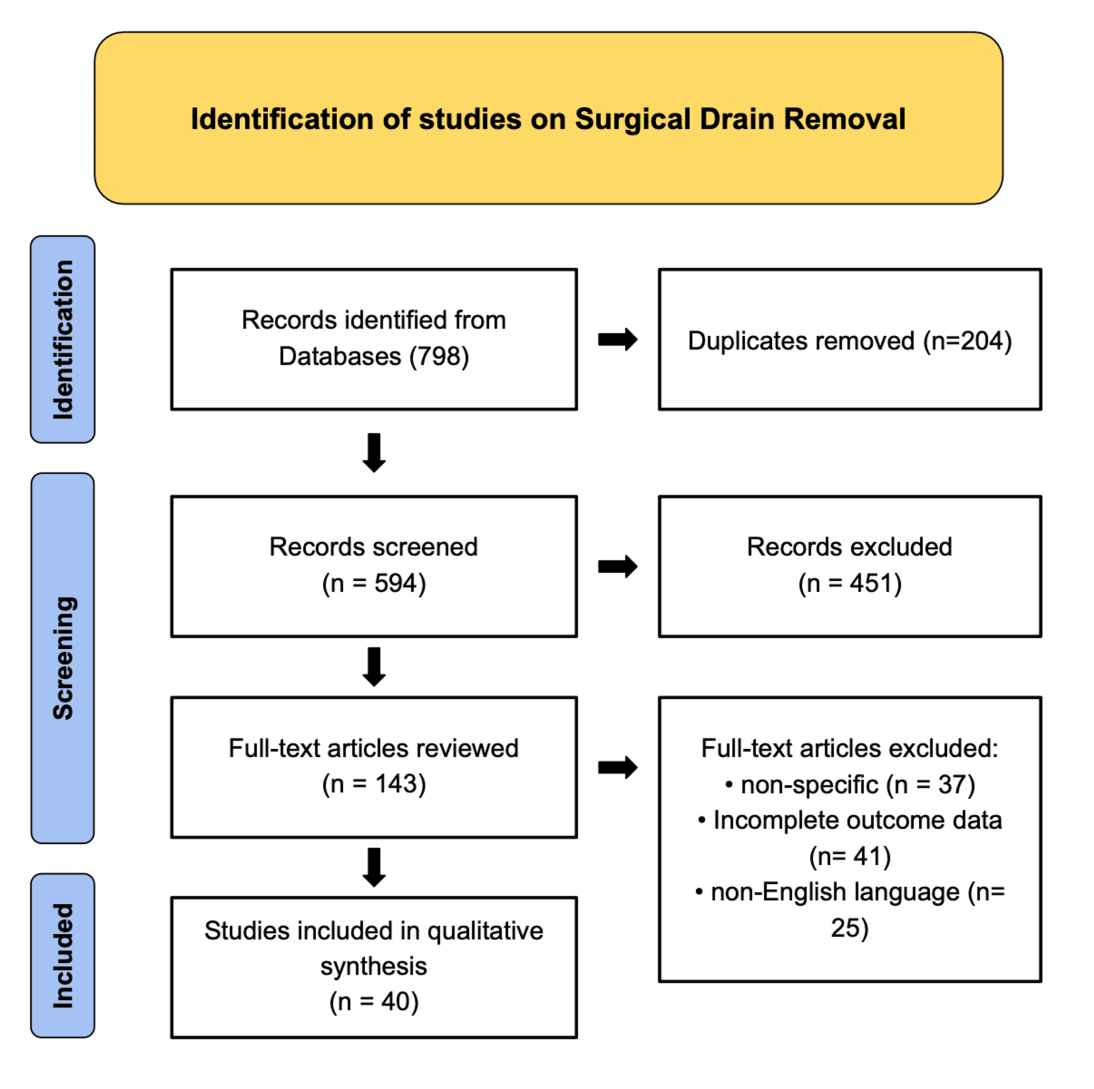
Preferred Reporting Items for Systematic Reviews and Meta-Analyses (PRISMA) Flow Diagram of Study Selection for Surgical Drain Removal Pertaining to Review of Timing Criteria, and Evidence-Based Practices. Flowchart illustrating the systematic identification, screening, eligibility, and inclusion process for studies evaluating surgical drain removal. A total of 798 records were identified through database searching, with 204 duplicates removed. After screening 594 records, 451 were excluded based on titles and abstracts. Of the 143 full-text articles reviewed, 103 were excluded for reasons including non-specific focus (n = 37), incomplete outcome data (n = 41),  and non-English language (n = 25). Ultimately, 40 studies were included in the qualitative synthesis.

A comprehensive literature search was conducted using PubMed, MEDLINE, and Google Scholar to identify English-language studies published between 1990 and 2025 examining surgical drain placement, management, and removal. The review process adhered to Preferred Reporting Items for Systematic Reviews and Meta-Analyses (PRISMA) guidelines (as depicted in Figure 1). Search terms and Boolean combinations included “surgical drain,” “drain removal,” “postoperative drainage,” “drain output,” “enhanced recovery after surgery (ERAS),” “drain management protocols,” “biochemical drain analysis,” “drain duration,” “seroma prevention,” “anastomotic leak detection,” and “postoperative complications.” Reference lists from relevant systematic reviews, meta-analyses, and society guidelines were manually screened to identify additional eligible studies.

Eligible studies included randomized controlled trials, prospective and retrospective cohort studies, meta-analyses, and expert consensus statements evaluating indications, timing, criteria, or outcomes related to surgical drain removal across general, hepatopancreaticobiliary, gastrointestinal, colorectal, thoracic, orthopedic, neurosurgical, and reconstructive procedures. Exclusion criteria comprised non-English publications, single-patient case reports, animal studies, and investigations without defined drain management protocols or measurable outcomes.

Four independent reviewers screened, extracted, and appraised eligible studies, blinded to each other’s selections to minimize bias and enhance objectivity. Each reviewer independently compiled information into standardized data extraction sheets and synthesized results based on the available evidence. Following individual review, findings were cross-verified and integrated to produce a unified qualitative narrative synthesis of the literature.

Extracted data (as depicted in Table 1) were organized into thematic domains, including output-based thresholds, biochemical markers (e.g., amylase, bilirubin, creatinine), timing-based removal strategies, procedure-specific variations, and postoperative complication outcomes such as infection, seroma, fistula, or abscess formation. Priority was given to high-level evidence from meta-analyses, randomized controlled trials, and professional society recommendations to ensure methodological rigor and comprehensive coverage.

**Table 1:** Summary of Key Literature on Surgical Drain Use and Timing of Removal. This table summarizes studies on surgical drain use, removal timing, and outcomes across various surgical specialties. It includes study design, patient population, surgical context, main findings, and clinical implications, highlighting evidence on early drain removal, selective versus routine placement, and innovations in drain management [1-38]. RCT: randomized controlled trial, SSI: surgical site infection, LOS: length of stay, CXR: chest X-ray, HPB: hepatopancreatobiliary, NSQIP: National Surgical Quality Improvement Program, ERAS: Enhanced Recovery After Surgery

Author (Year)	Study Design / Population	Surgical Context	Main Findings	Key Implications
Chen CF et al. (2016) [1]	Retrospective, 150 pts	Breast reconstruction	Infection risk correlated with drain duration, not daily output	Early removal lowers infection risk
Ssenyondo EK et al. (2013) [2]	RCT, 60 pts	Thyroidectomy	No significant difference in hematoma or seroma with vs. without drains	Routine drains unnecessary in thyroid surgery
Cui P et al. (2024) [3]	Comparative study, 200 pts	Lumbar fusion	Early removal (≤48h) showed no increased complications	Supports earlier drain removal criteria
Ishinuki T et al. (2023) [4]	Systematic review & meta-analysis	Abdominal surgery	Subcutaneous drains did not significantly reduce SSI	Routine subcutaneous drains may be avoided
Groothoff MS et al. (2025) [5]	Scoping review, 50+ studies	Emergency general surgery	Limited evidence supporting prophylactic drains	Drain use should be selective, not routine
Brown MD et al. (2004) [6]	Double-blind RCT, 100 pts	Lumbar spine surgery	Drains did not affect infection or hematoma rates	Routine use unnecessary
Meyerson JM (2016) [7]	Narrative review	Surgical history	Overview of evolution of common surgical drains	Highlights origins of modern drainage principles
Bean KJ (1983) [8]	Historical review	General surgery	Describes evolution from sump to suction drains	Contextualizes current drain technology
Cheng Y et al. (2016) [9]	Cochrane review	Pancreatic surgery	Routine prophylactic drainage showed no mortality benefit	Supports selective drain placement
Kushner B et al. (2021) [10]	Retrospective, 200 pts	Abdominal wall reconstruction	Early removal didn’t increase SSI	Encourages early drain removal protocols
Yoon SJ et al. (2021) [11]	Retrospective, 120 pts	Pancreatoduodenectomy	Early removal reduced complications and LOS	Early removal is safe and promotes recovery
Rowbottom RD et al. (2024) [12]	Retrospective	Cardiothoracic surgery	Routine CXR post drain removal rarely altered management	Routine imaging post-removal unnecessary
Wu AGR et al. (2023) [13]	Systematic review & meta-analysis	Pancreatic resection	Early removal reduced fistula and LOS	Early removal favored
Kowal M et al. (2022) [14]	Systematic review	HPB surgery	Monitoring output improved detection but not outcomes	Quantitative monitoring may not change results
Kim C et al. (2023) [15]	Prospective, 80 pts	Spine surgery	Drain tip cultures not predictive of SSI	Routine cultures unnecessary
Chua C et al. (2022) [16]	Retrospective	Plastic surgery	24h output and POD not reliable for removal timing	Drain removal should be individualized
Kent MS et al. (2024) [17]	Expert consensus	Thoracic surgery	Standardized drain management after lobectomy	Promotes uniform evidence-based practice
Bohorquez D et al. (2022) [18]	Retrospective	Head & neck surgery	Output volume poorly predicted safe removal	Clinical judgment superior to numeric criteria
Yoshimura K et al. (2023) [19]	Retrospective	Colorectal surgery	Early removal safe when drainage ≤100 mL/day	Supports early removal threshold
Roebker JA et al. (2023) [20]	Practical review	Thoracic surgery	Summarized modern management and complications	Provides updated chest tube guidance
Yu H (2011) [21]	Review	Pulmonary	Comprehensive overview of pleural drainage	Educational reference for chest drain care
Rekavari SG et al. (2024) [22]	Review	General surgery	No universal benefit from prophylactic drains	Encourages selective use based on procedure
Li T et al. (2023) [23]	Meta-analysis	Pancreatic surgery	Early removal reduced fistula and LOS	Confirms early removal safety
Qualliotine JR et al. (2020) [24]	Case report	Head & neck surgery	Self-removing drains feasible via telehealth	Supports remote postoperative management
Bray JO et al. (2023) [25]	Prospective	Hernia repair	At-home drain removal safe and feasible	Enables outpatient self-care
Meckler A et al. (2025) [26]	Pilot study	General surgery	AI-assisted drain analysis improved monitoring	Introduces digital drain technology
Durai R & Ng PCH (2010) [27]	Review	General surgery	Described drain types, uses, complications	Classic educational reference
Yee EJ et al. (2021) [28]	NSQIP database study	Colorectal liver metastases	Drain use variable, no outcome difference	Selective drain use recommended
Livingston AJ et al. (2020) [29]	Review	ENT/Neurosurgery	Practical overview of lumbar drain management	Guides safe clinical use
Goldaracena N et al. (2022) [30]	Systematic review	Liver transplantation	Early removal reduced LOS and improved recovery	Supports ERAS protocols
Majercik S et al. (2025) [31]	RCT	Rib fracture fixation	Thoracostomy reduced fluid and complications	Supports routine thoracic drainage in select cases
Miao C et al. (2025) [32]	Cochrane review	Pancreatic surgery	No survival or morbidity benefit from routine drainage	Reaffirms selective drainage
Afoke J et al. (2014) [33]	Prospective	Thoracic surgery	Digital drains shortened removal time	Supports digital monitoring use
Harris T et al. (2011) [34]	Retrospective	Head & neck surgery	Safe removal typically at 24–48h	Early removal safe once output minimal
Sepehripour AH et al. (2012) [35]	Retrospective	Cardiothoracic surgery	Routine post-removal CXR rarely changed management	Eliminating unnecessary imaging
Miller C et al. (2017) [36]	Retrospective	Neurosurgery	Hemorrhage risk increased with coagulopathy	Caution with anticoagulated patients
French DG et al. (2016) [37]	Review	Thoracic surgery	New tech and protocols improved outcomes	Highlights evidence-based postoperative care
Lewis A et al. (2017) [38]	Retrospective	Neurosurgery	Antibiotic prophylaxis reduced infection with drains	Supports targeted prophylaxis

​Drain removal criteria: volume-based, time-based, and biochemical-guided approaches

The optimal timing of surgical drain removal is essential to balance the benefits of fluid evacuation against the risks of prolonged indwelling devices. Historically, two predominant criteria have guided removal decisions: volume-based thresholds and time-based cutoffs.

Volume-Based Criteria

**Figure 2:**
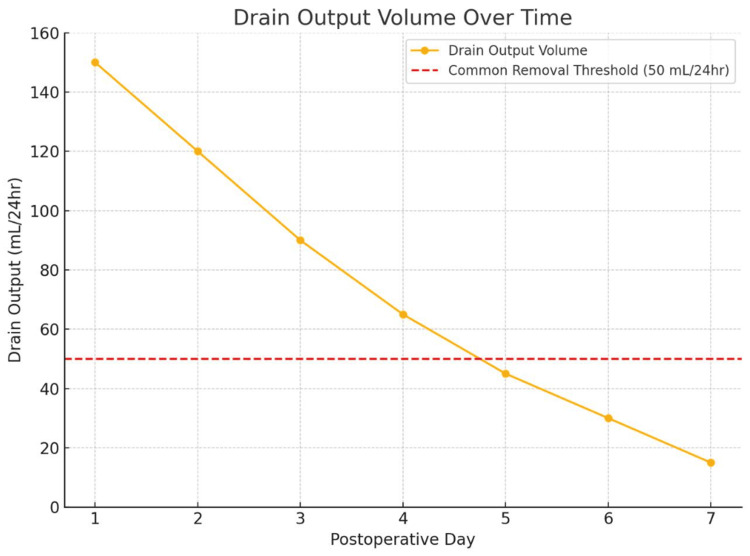
Drain Output Volume Over Time with Common Removal Threshold. This figure illustrates a typical postoperative decline in drain output volume over the first seven days following surgery. The red dashed line represents a commonly used removal threshold of 50 mL/24 hours, a criterion often applied in head and neck, breast, and abdominal surgeries [15,16,18]. When output falls below this threshold and no signs of active bleeding, bile, or infection are present, early removal is generally considered safe. This visual highlights the convergence of volume-based and time-based approaches to drain management and supports the growing trend toward early, evidence-based drain removal to minimize complications and enhance recovery. The figures presented were generated and designed by the authors for this study.

Volume thresholds (as depicted in Figure 2) remain a commonly employed strategy, with many protocols recommending removal when drainage falls below 30-50 mL over 24 hours, provided there is no evidence of infection, bile, or hemorrhage. This approach has been widely used in breast, head and neck, and abdominal surgeries and has demonstrated safety and efficacy in reducing seroma and infection risks [15-17]. In colorectal and hepatobiliary surgery, output under 50 mL/day without signs of active bleeding or leakage is often used as a cutoff, correlating with reduced surgical site infection (SSI) rates [18,19].

Time-Based Criteria

Alternatively, a fixed postoperative timeline is sometimes employed, particularly in pancreatic, thyroid, and spine surgeries. For example, recent trials suggest that removing drains by postoperative day three in low-risk pancreatic surgery patients significantly reduces the incidence of pancreatic fistula, shortens hospital stays, and improves recovery [20]. Similarly, in head and neck surgery, some evidence supports safe removal within 48 hours even before output falls below traditional thresholds [17].

Biochemical Marker-Guided Criteria

Advances in postoperative monitoring have introduced the use of biochemical analysis of drain fluid to guide timing. In pancreatic surgery, the International Study Group on Pancreatic Fistula (ISGPF) recommends removal when drain amylase levels on postoperative day one are less than three times the upper normal limit of serum amylase, a finding linked to reduced fistula formation [21,20]. Similar approaches are emerging in hepatobiliary and thyroid surgery, with drain creatinine or bilirubin levels used in select cases to rule out bile or urine leaks [20,19].

Individualized Clinical Judgment

Despite these frameworks, a growing consensus supports individualized drain management that incorporates patient-specific risk factors, intraoperative findings, and surgeon experience. Studies increasingly discourage rigid adherence to single-parameter thresholds, favoring a holistic evaluation of the patient’s clinical trajectory, wound healing status, and comorbidities [15,22,23].

Procedure-Specific Protocols

**Figure 3:**
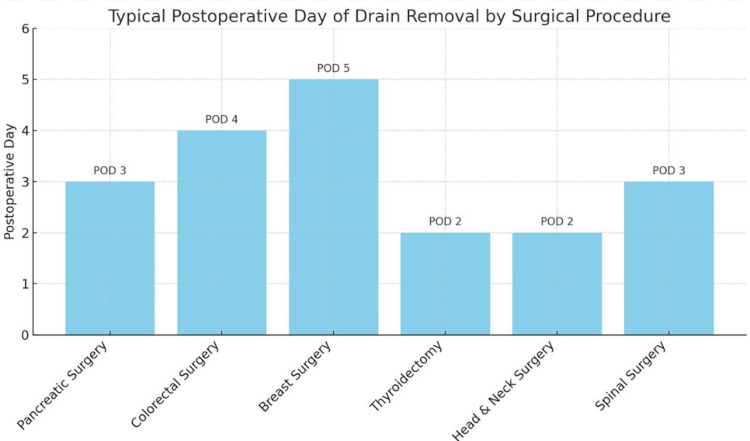
Common Drain Removal Timelines by Surgical Procedure. This figure summarizes typical postoperative days (POD) when surgical drains are removed across various surgical specialties. For example, pancreatic and spinal surgeries often allow for safe removal by POD 3, whereas breast and colorectal surgeries may require longer observation periods. These timelines reflect evidence-based trends reported in the literature [15-19,23,24]. The figures presented were generated and designed by the authors for this study.

Surgical specialty also influences criteria. As depicted in Figure 3, in head and neck surgery, drains are often removed based on output regardless of timing [16,17], while in gastrointestinal surgery, timing protocols increasingly favor early removal or complete avoidance of drains [24,25]. In cardiac surgery, prolonged use is often avoided due to risks of bleeding and tissue trauma, with careful monitoring of output color and volume guiding decisions [26].

Procedure-specific variations

Surgical drain management in general surgery encompasses a range of procedures most notably gastrointestinal, hepatobiliary, and pancreatic operations where the decision to place, maintain, or remove drains must balance fluid evacuation benefits with the risks of infection, delayed healing, and patient discomfort. Evidence increasingly supports tailored approaches that avoid routine drainage when not clinically indicated and promote early removal when drains are used.

Gastrointestinal Surgery

In gastrointestinal procedures, the role of prophylactic drains has been re-evaluated in light of multiple studies showing no significant benefit for many standard operations. For hepatic resections, colonic and rectal resections with primary anastomosis, and appendectomy across all stages of appendicitis, routine drainage has not been shown to reduce postoperative complications and may increase infection risk or impair wound healing, particularly in patients with cirrhosis or compromised hepatic function [24]. Routine use is generally discouraged, except in operations with high anastomotic leak risk such as esophagectomy or total gastrectomy where prophylactic drainage may help detect and manage potentially life-threatening leaks [25]. In these high-risk cases, closed suction systems are preferred, with individualized removal criteria based on intraoperative findings and patient-specific risk factors.

Pancreatic Resections

Pancreatic surgery presents unique challenges, with postoperative pancreatic fistula (POPF) remaining the most feared complication. Prospective data support early removal often by postoperative day three in low-risk patients which is associated with reduced POPF rates, fewer abdominal and pulmonary complications, and shorter hospital stays [20]. Risk stratification incorporates gland texture, pancreatic duct size, and nutritional status. Individualized drain management with close monitoring is essential, particularly as delayed removal in high-risk patients may be warranted to monitor for leakage.

Hepatobiliary Surgery

In hepatobiliary operations, particularly liver resections, evidence suggests that routine drainage offers no clear benefit in uncomplicated cases. However, selective drainage remains appropriate for extensive resections, biliary-enteric anastomoses, or when bile leakage is a concern. Early removal, typically within three to five days when output is low and non-bilious, has been associated with decreased surgical site infection rates and shorter hospitalization [19]. For cholecystectomy open or laparoscopic drains are rarely indicated except in cases of significant bile spillage, severe inflammation, or when conversion from laparoscopic to open surgery raises suspicion of postoperative collections.

Abdominal Wall and Hernia Surgery

In ventral hernia repair and other major abdominal wall reconstructions, routine drain placement is not universally required, but may be used to reduce seroma or hematoma formation in cases involving large dead spaces or significant subcutaneous dissection. When placed, early removal is recommended to minimize infection risk, as prolonged drainage offers little additional benefit [27].

Drain Use Across Surgical Specialties

The utility of prophylactic drains varies by procedure. In subcutaneous surgeries, drains reduce hematoma after breast biopsy and seroma following axillary lymph node dissection, but routine use in cesarean sections, abdominal wall closures, femoral wounds, and joint arthroplasties shows no meaningful reduction in complications, even in high-risk obese patients, supporting selective rather than routine use [27]. Similarly, in spinal surgery, closed-suction drains do not significantly prevent hematoma, seroma, or infection after procedures such as cervical fusion, lumbar decompression, or scoliosis correction, and extending antibiotics beyond 24 hours offers no additional benefit [28,29]. In head and neck surgery, earlier drain removal, even within 48 hours, appears safe and may reduce discomfort, challenging rigid output-based criteria [16,17].

In urological and cardiac surgery, drain and prophylaxis strategies are increasingly individualized. Minimally invasive urologic procedures, including laparoscopic partial nephrectomy and robot-assisted prostatectomy, question routine drain use, with decisions guided by bleeding risk, procedure complexity, and patient factors [30-32]. Cardiac surgery patients require careful balancing of thromboembolism and bleeding risk; mechanical prophylaxis is preferred initially in high-risk bleeding patients, with pharmacologic therapy added once hemostasis is secured. Minimizing manipulation of mediastinal drains optimizes recovery without improving outcomes [32,33].

In summary, surgical drain management must be adapted to the specific risks and requirements of each procedure to optimize patient outcomes. Table 2 provides a detailed overview of the key considerations and current best practices across the surgical disciplines discussed, serving as a practical guide for individualized drain management strategies.

**Table 2:** Summary of Surgical Drain Management Across Various Disciplines. This table summarizes current recommendations for surgical drain use, removal criteria, and key considerations across multiple surgical specialties. It highlights procedure-specific variations in the necessity and timing of drain removal, reflecting evolving evidence aimed at optimizing patient outcomes and minimizing complications [16-19,24-27,32]. POPF: postoperative pancreatic fistula, POD: postoperative days, ERAS: Enhanced Recovery After Surgery, SSI: surgical site infection The Tables presented were generated and designed by the authors for this study.

Specialty / Procedure	Drain Use Indication	Removal Criteria	Key Considerations
Gastrointestinal Surgery	Selective use in high-risk anastomoses (e.g., esophagectomy, total gastrectomy). Avoid routine use in uncomplicated colorectal and appendectomy cases.	<50 mL/24h, non-bilious/non-enteric, no signs of leak	Closed suction drains are preferred; avoid in low-risk cases to reduce infection risk.
Pancreatic Surgery	Used to monitor for POPF in resections. Avoid routine use in low-risk distal pancreatectomy.	POD 3 removal if amylase normal and output minimal	Risk stratify based on gland texture, duct size, and nutritional status.
Hepatobiliary Surgery	Selective drainage for extensive resections, biliary-enteric anastomoses, bile leak concern.	3–5 days if output low and non-bilious	Rarely indicated in standard cholecystectomy unless bile spillage or severe inflammation.
Colorectal Surgery	Selective use in high pelvic anastomoses; avoid routine use in primary anastomosis.	<50 mL/24h with no clinical leak signs	ERAS protocols recommend omission in low-risk resections.
Abdominal Wall / Hernia Surgery	Consider a large dead space or significant subcutaneous dissection.	Early removal when drainage low and serosanguinous	Prolonged drainage increases SSI risk; limited benefit beyond early postoperative period.

Complications of prolonged drain use

While surgical drains remain essential in general surgery for fluid evacuation, prevention of fluid collections, and reduction of dead space, prolonged retention can significantly increase postoperative morbidity. In abdominal procedures, including hepatobiliary, colorectal, and pancreatic surgeries, extended drain placement is associated with risks such as drain malfunction, blockage, delayed wound healing, tissue irritation, and SSI [34-36]. Prolonged presence also predisposes to bacterial colonization of the drain tract, potentially resulting in intra-abdominal abscesses, anastomotic leakage, or wound breakdown.

Monitoring and Patency

Daily monitoring of drain output, color, and character is essential. In general surgery, low output does not always equate to safe removal; partial occlusion by fibrin or necrotic debris can mask ongoing leakage, particularly in pancreatic and biliary drains. Failure to identify such blockages can delay necessary intervention. Maneuvers such as drain “stripping” or “milking” may restore patency, reducing the risk of partial obstruction.

Infection and Local Tissue Effects

Extended drain presence, especially beyond postoperative day five to seven without clinical indication, serves as a nidus for infection and prolongs recovery. Evidence from hepatobiliary and colorectal surgery demonstrates higher SSI rates when drains are left beyond a week [35,36]. The foreign body effect can also precipitate local inflammatory changes, resulting in pain, erythema, seroma, and impaired wound healing [37]. Persistent irritation may lead to ulceration or skin necrosis, lengthening hospital stay.

Fistula Formation

Fistula formation, particularly in pancreatic and gastrointestinal surgery, remains a major concern. Prolonged drainage in the presence of high-amylase content fluid has been linked to pancreatic fistulas, which delay closure of anastomotic sites and increase the need for reoperation [38]. Similarly, biliary fistulas may arise when drains create or maintain an unnatural tract for fluid egress.

Hemorrhage Risk

Hemorrhage, while uncommon, may occur in highly vascular surgical fields such as hepatic resections. Negative pressure from suction drains can disrupt fragile capillaries, leading to bleeding or hematoma formation [28,34]. Proper drain positioning, secure fixation, and early removal when clinically appropriate are essential in minimizing these risks.

Emerging trends and recommendations

Contemporary trends in general surgery increasingly favor early drain removal, supported by evidence that prolonged drain use may elevate the risk of SSI, intra-abdominal abscesses, and fistula formation without improving outcomes [23,22]. ERAS protocols, widely adopted in gastrointestinal, hepatobiliary, and colorectal procedures, promote standardized perioperative care and advocate for minimizing or avoiding prophylactic drains unless clearly indicated [24].

In abdominal surgery, controlled studies suggest that removal once output falls below 50 mL over 24 hours in the absence of infection or active bleeding is safe and may reduce morbidity [18,19]. For example, in colorectal resections, prolonged drainage beyond this threshold has not shown benefit in preventing anastomotic leak but has been linked to increased SSI risk [19]. In pancreatic resections, early removal by postoperative day three in low-risk patients correlates with reduced fistula rates and shorter hospital stays [38].

These findings reflect a shift toward individualized, evidence-based drain management, tailoring timing to patient-specific factors such as intraoperative findings, output characteristics, and overall risk profile. When drains are indicated, ongoing reassessment is essential, and non-essential drains should be removed promptly to facilitate recovery.

Technological and procedural innovations are also influencing drain management. Digital drain systems with continuous monitoring, improved seal integrity, and lower risk of retrograde infection are being evaluated in abdominal surgery. Telemedicine-based follow-up, piloted during the COVID-19 pandemic, has allowed safe home drain monitoring and removal in select low-risk patients, reducing hospital visits without compromising safety [33,35].

Overall, the modern approach in general surgery emphasizes minimizing prophylactic drain use, adopting strict removal criteria, and integrating drain management into ERAS pathways. Continued high-quality research in procedure-specific contexts will be critical to refining guidelines, standardizing practices, and improving patient outcomes across the spectrum of general surgical procedures [36,38].

## Conclusions

In general surgery, surgical drains remain an important tool for reducing postoperative fluid collections, minimizing dead space, and preventing early complications. Their benefits, however, must be weighed against the risks of prolonged retention, which can include infection, abscess formation, fistula development, bleeding, and delayed wound healing. Best practices increasingly emphasize early, criteria-based removal guided by output, fluid characteristics, and clinical judgment. Tailoring decisions to operative findings and patient factors, such as selective placement in high-risk anastomoses and avoidance in low-risk cases, ensures safer and more individualized management.

The figures presented in this review illustrate both the typical decline in drain output over the postoperative period and procedure-specific timing benchmarks, reinforcing the role of objective monitoring in guiding removal decisions. By integrating volume thresholds, biochemical markers, and individualized clinical judgment, surgeons can reduce morbidity, improve patient comfort, and facilitate earlier discharge.

Enhanced recovery pathways have further supported this approach by promoting minimal drain use, earlier removal, and integration with other perioperative strategies to expedite recovery. Looking ahead, standardizing removal protocols, aligning practices across institutions, and refining patient-centered strategies will be key to improving outcomes. By combining objective measures with individualized decision-making, surgeons can reduce complications, enhance patient comfort, and support earlier discharge.
